# Recurrent *SLCO1B1* and *SLCO1B3* mutations identified in three patients with Rotor syndrome

**DOI:** 10.3389/fmed.2025.1630360

**Published:** 2025-08-18

**Authors:** Chenyu Zhao, Hui Huang

**Affiliations:** ^1^Department of Gastroenterology, Henan Provincial People’s Hospital, Zhengzhou University People’s Hospital, Zhengzhou, China; ^2^Department of Medical Genetics, The Second Xiangya Hospital, Central South University, Changsha, China

**Keywords:** Rotor syndrome, *SLCO1B1*, *SLCO1B3*, mutation, hyperbilirubinemia

## Abstract

**Background:**

Rotor syndrome is a rare genetic disease inherited in an autosomal digenic recessive manner. It is caused by pathogenic mutations in both *SLCO1B1* and *SLCO1B3* genes, and characterized by predominantly conjugated hyperbilirubinemia.

**Methods:**

Three Chinese patients clinically diagnosed with Rotor syndrome were included. Mutations in *SLCO1B1/3* genes were identified using whole-exome sequencing.

**Results:**

They all carried the same homozygous c.1738C>T mutation in *SLCO1B1* and the c.481+22insLINE variant in *SLCO1B3*.

**Conclusion:**

This study established a genetic diagnosis for the three patients and contributed to finding hotspot mutations in Rotor syndrome.

## 1 Introduction

Rotor syndrome (RS, OMIM*237450) is a rare and benign genetic disease characterized by low-grade, chronic or fluctuating, predominantly conjugated hyperbilirubinemia. It has no other features of hepatobiliary disorder (1, 2). The prevalence of RS is unknown but is very low (<1:1,000,000) (3). First described by Rotor and Florentin (4) in 1948, it is inherited in an autosomal recessive digenic manner. Biallelic pathogenic mutations in solute carrier organic anion transporter family member 1B1 (*SLCO1B1*) and *SLCO1B3* genes cause RS. Organic anion transporting polypeptide 1B1 (OATP1B1) and OATP1B3 are encoded by *SLCO1B1* and *SLCO1B3* genes, respectively. They serve as transporters for hepatic uptake of conjugated bilirubin. Inactivation of both proteins together leads to RS, which does not affect life expectancy and usually requires no treatment (1). To date, 51 mutations in *SLCO1B1* and 30 variants in *SLCO1B3* have been described in the Human Gene Mutation Database (HGMD; http://www.hgmd.cf.ac.uk/ac/index.php). In the present study, we reported three patients with RS and tested two disease-causing mutations: *SLCO1B1* (NM_006446.5): c.1738C>T (p.R580*) and *SLCO1B3* (NM_019844.4): c.481+22insLINE.

## 2 Materials and methods

### 2.1 Patients and ethics

Three unrelated Chinese patients (numbered 1–3) were enrolled in this study. Patients 1, 2, and 3 were aged 14, 16, and 20 years, respectively. They were clinically diagnosed as RS without other clinical comorbidities. They intermittently took S-adenosylmethionine or diammonium glycyrrhizinate for treatment. All patients or their guardians signed the written informed consent forms. This study was approved by the ethics committee of the Second Xiangya Hospital of Central South University.

### 2.2 Variant analysis

Genomic DNA was isolated from peripheral blood by the Blood gDNA Miniprep Kit (Hangzhou Beiwo Meditech Co., Ltd., Hangzhou, China). Whole-exome sequencing (WES) was performed on the three probands using the MGISEQ-2000 platform (MGI Tech Co., Ltd., Shenzhen, China). WES and basic bioinformatics analyses, including read mapping and variant detection, were performed by AmCare Genomics Lab Limited (Guangzhou, China).

The methods for filtering WES data are as follows: (1) Variants from the databases (1000G, ExAC, esp6500, gnomAD) with a minor allele frequency of >5% were excluded. (2) Variants in untranslated regions and synonymous mutations were excluded. (3) The candidate pathogenetic variants in bilirubin metabolism-related genes were retained. (4) The interpretation of mutation pathogenicity was guided by the American College of Medical Genetics and Genomics (ACMG) guideline (5). The potential variant from WES was validated by Sanger sequencing.

## 3 Results

**Figure 1:**
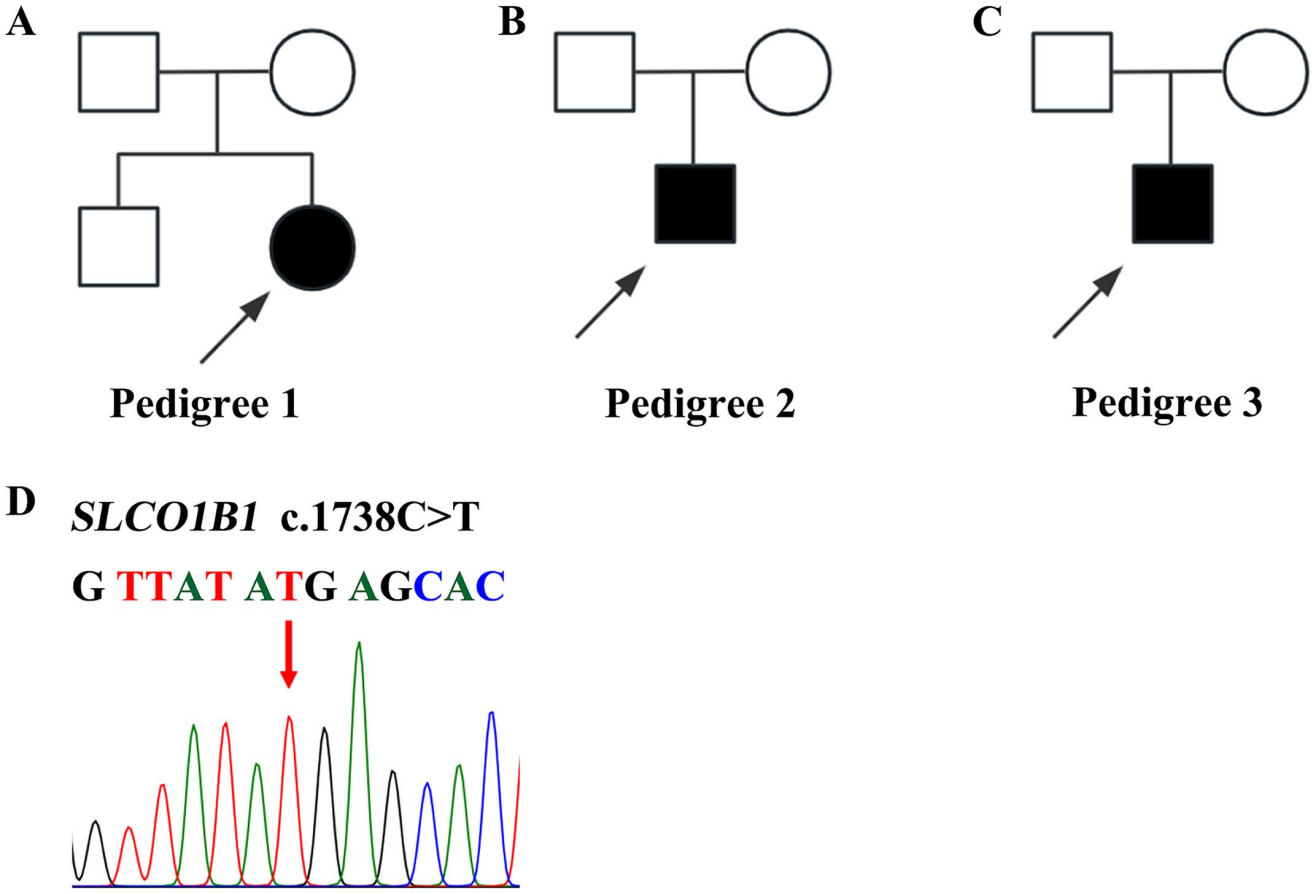
Pedigrees and variants of *SLCO1B1* in patients with Rotor syndrome. **(A–C)** Three pedigrees affected with Rotor syndrome. **(D)** Homozygous c.1738 C>T *SLCO1B1* mutation identified in three probands.

This study included two male and one female patient, aged from 14 to 20 years. They were born to nonconsanguineous parents (Figures 1A–C) and presented with mild intermittent jaundice. The liver function test only showed predominantly conjugated hyperbilirubinemia. No abnormalities were observed in viral serologies (HBV and HCV), hemolysis test, coagulation function, autoimmune liver disease-associated antibodies, immunoglobulin G, serum ceruloplasmin testing, or abdominal ultrasound examination. The major clinical manifestations of the three patients with Rotor syndrome are summarized in Table 1. The WES indicated that all patients harbored the same homozygous c.1738C>T mutation in *SLCO1B1* (Figure 1D) and c.481+22insLINE variant in *SLCO1B3*.

**Table 1:** Clinical characteristics and mutations in *SLCO1B1/3*.

Patient	1	2	3
Gender	Female	Male	Male
Age	14	16	20
**Symptoms**			
Intermittent jaundice	+	+	+
**Laboratory data**			
Hb (g/L)	126	149	152
ALT/AST (U/L)	11.9/23.6	15.8/17.7	31.7/18.5
TBIL/DBIL (μmol/L)	76.1/48.3	108.9/70.7	129.2/100.6
GGT (U/L)	14	12.7	27.3
Viral serologies (HBV and HCV)	—	—	—
Hemolysis test	—	—	—
Ultrasound examination of the liver, gallbladder, pancreas, and spleen	N	N	N
**Genetic testing**			
Mutation in *SLCO1B1*	c.1738C>T	c.1738C>T	c.1738C>T
Mutation in *SLCO1B3*	c.481+22insLINE	c.481+22insLINE	c.481+22insLINE

Hb, hemoglobin (normal range: 130–170 g/L); ALT, alanine aminotransferase (normal range: 9–50 U/L); AST, aspartate aminotransferase (normal range:15–40 U/L); TBIL, total bilirubin (normal range: 3.4–17.1 μmol/L); DBIL, direct bilirubin (normal range: normal range: 0–6 μmol/L); GGT, gamma-glutamyl transpeptidase (normal range: 10–60 U/L); HBV, hepatitis B virus; HCV, hepatitis C virus; NA, not available; N, normal.

## 4 Discussion

**Figure 2:**
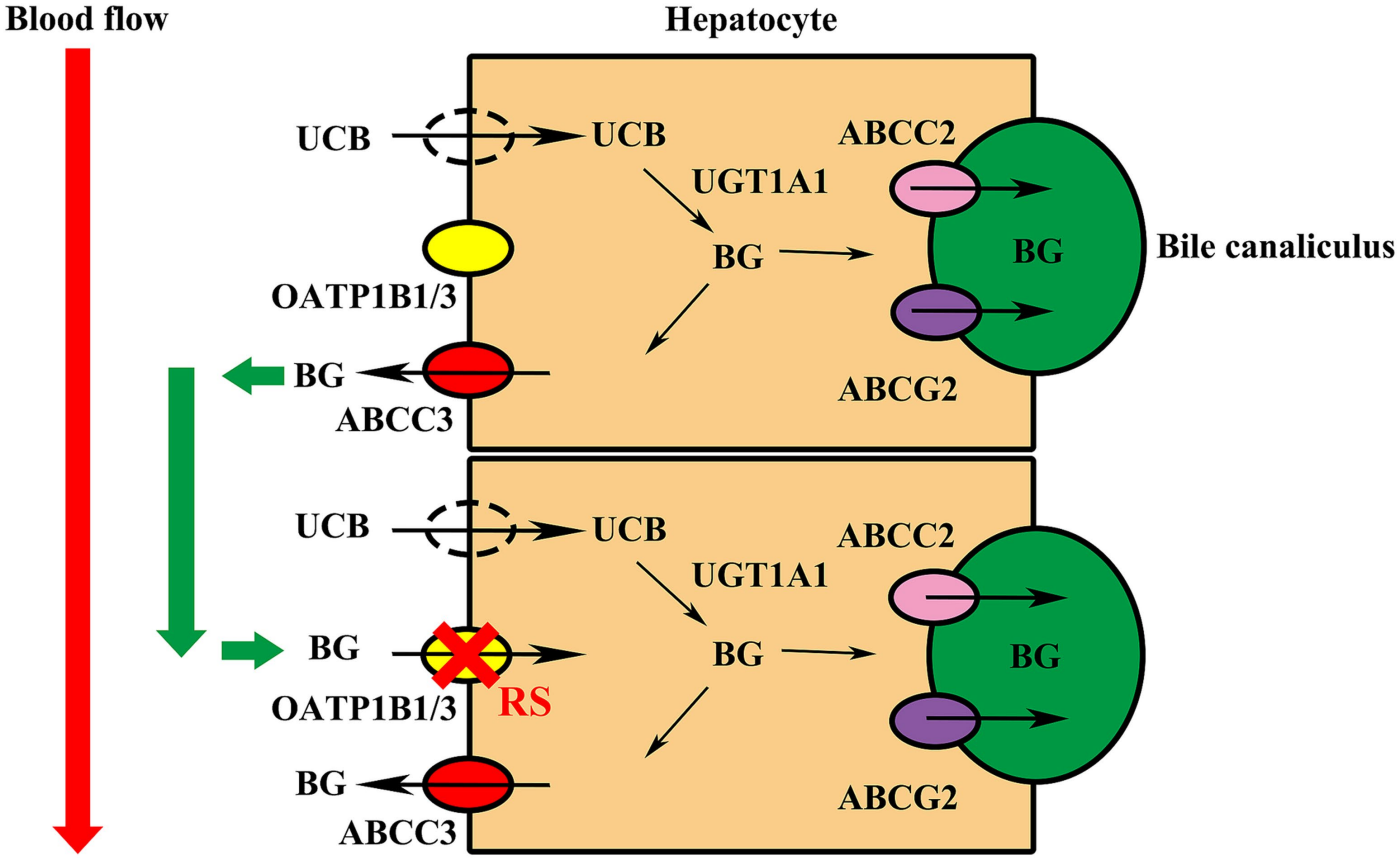
Schematic view of bilirubin transport by the hepatocyte in Rotor syndrome. In hepatic metabolism, UGT1A1 catalyzes the conversion of UCB to water-soluble BG within the endoplasmic reticulum. The generated BG is secreted into bile by ABCC2 and ABCG2 transporters, while a substantial fraction is rerouted to the bloodstream via ABCC3. Downstream hepatocytes can uptake BG from the circulation through OATP1B1/3 transporters. In RS, the absence or dysfunction of OATP1B1/3 transporters disrupts hepatic uptake of BG. UGT1A1, uridine diphosphate glucuronosyltransferase 1A1; UCB, unconjugated bilirubin; BG, bilirubin glucuronides; RS, Rotor syndrome.

OATP1B1/3 are expressed in the hepatocyte basolateral membrane, which are also called SLCO1B1/3. They uptake endogenous substances, such as conjugated bilirubin, bile acids (BAs), eicosanoids, prostaglandins, and hormones. Unconjugated bilirubin (UCB) enters hepatocytes through passive diffusion and/or transporters, which may include OATP1B1/3. Uridine-diphospho glucuronosyl transferase 1A1 (UGT1A1) catalyzes the conversion of UCB to bilirubin glucuronides (BG) in the endoplasmic reticulum. BG is secreted into bile by ABCC2 and ABCG2. A substantial fraction of BG is rerouted by ABCC3 to the blood. It can be taken up by downstream hepatocytes via OATP1B1/3 transporters (2). In RS, the absence or dysfunction of the OATP1B1/3 may disrupt the uptake of BG (Figure 2), which causes predominantly conjugated hyperbilirubinemia.

The accurate diagnosis of RS is of paramount clinical importance, as it directly influences pharmaceutical safety by preventing unwarranted exposure to drugs that rely on functional OATP1B1/3 transporters for hepatic uptake and systemic clearance. The dysfunction of OATP1B1/3 transporters can profoundly impact numerous drug metabolisms, particularly statins, ezetimibe, methotrexate, irinotecan, cabazitaxel, sunitinib, and sartans (3, 6–8). They could exhibit significantly increased systemic exposure in RS patients. After a confirmed diagnosis of RS, clinicians can proactively select alternative drugs with minimal OATP dependency and implement therapeutic drug monitoring (TDM) for high-risk agents.

The c.1738C>T is a nonsense variant in *SLCO1B1*, which was very strong evidence of pathogenicity (PVS1). The mutation is located in a mutational hotspot (PM1). It has been reported in multiple clinical cases of RS (6, 9–12). Prediction software, specifically MutationTaster (13), predicted a deleterious effect of the variant (PP3). Therefore, according to ACMG guidelines, the variant is classified as a pathogenic mutation.

The c.481+22insLINE mutation in *SLCO1B3* is a long-interspersed element (LINE) insertion variant about 6.1 kb in size. It could affect normal editing of mRNA and cause abnormal skipping of exons (PS3) (10). It is a common mutation of RS in Asian populations (PM1) (10). The variant is not found in either the 1000G or EXAC databases (PM2). The patients carried both the homozygous c.1738C>T variant in *SLCO1B1* and the c.481+22insLINE mutation in *SLCO1B3*. The findings were consistent with the digenic recessive pattern of RS (PM3). Therefore, the variants were also classified as a pathogenic mutation.

This study provided evidence that the detected genetic mutations in *SLCO1B1* and *SLCO1B3* are common in Rotor syndrome, which is consistent with previous research (6, 9–12, 14). However, precise information on the frequencies of c.1738C>T (*SLCO1B1*) and c.481+22insLINE (*SLCO1B3*) mutations is limited. There is insufficient data to assess its distribution in RS and the general population. In resource-limited settings across East Asia, targeted PCR assays for the two mutations might be considered as a screening method for RS; however, validation in larger sample sizes is required.

In conclusion, RS is a rare inherited disorder that causes predominantly conjugated hyperbilirubinemia. Genetic testing is a useful tool for efficient diagnosis. This study supported that c.1738C>T in *SLCO1B1* and c.481+22insLINE in *SLCO1B3* are common pathogenic mutations in the East Asian RS patients.

## Data Availability

The datasets for this article are not publicly available due to concerns regarding participant/patient anonymity. Requests to access the datasets should be directed to the corresponding author.
